# ChatGPT-4's Consistency, Specificity, and Inclusion of Behavior Change Techniques in Delivering Smoking Cessation Advice in Traditional Chinese: A Content Analysis

**DOI:** 10.1093/ntr/ntaf267

**Published:** 2025-12-24

**Authors:** Xiao Yun Xie, Min Jin Zhang, Zhi Jie Kelman Cheung, Tzu Tsun Luk, Man Ping Wang, Tai Hing Lam, Yee Tak Derek Cheung

**Affiliations:** School of Nursing, Li Ka Shing Faculty of Medicine, The University of Hong Kong, Hong Kong SAR, China; School of Nursing, Li Ka Shing Faculty of Medicine, The University of Hong Kong, Hong Kong SAR, China; Nursing Department, First Affiliated Hospital, Sun Yat-sen University, Guangzhou, China; School of Nursing, Li Ka Shing Faculty of Medicine, The University of Hong Kong, Hong Kong SAR, China; School of Nursing, Li Ka Shing Faculty of Medicine, The University of Hong Kong, Hong Kong SAR, China; Alice Lee Centre for Nursing Studies, Yong Loo Lin School of Medicine, National University of Singapore, Singapore; School of Nursing, Li Ka Shing Faculty of Medicine, The University of Hong Kong, Hong Kong SAR, China; School of Public Health, Li Ka Shing Faculty of Medicine, The University of Hong Kong, Hong Kong SAR, China; School of Nursing, Li Ka Shing Faculty of Medicine, The University of Hong Kong, Hong Kong SAR, China

## Abstract

**Introduction:**

While ChatGPT has shown promise in health domains, its application in smoking cessation, particularly in non-English contexts, remains underexplored. This study assessed the consistency, specificity, and inclusion of behavior change techniques in ChatGPT-4's advice for smoking-related queries in Traditional Chinese across three phases.

**Methods:**

ChatGPT-4 was accessed via Azure OpenAI Services (temperature: 0.7, Top P: 0.95). Phase I assessed consistency of 10 responses to identical smoking-related questions, measured using the Jaccard coefficient. Phase II evaluated response specificity using 12 smoking-related vignettes, tailored to age, readiness to quit, and nicotine dependence, through Jaccard distances and alignment to the 2008 U.S. Clinical Practice Guideline. In phase III, responses to 20 detailed smoking-related vignettes were analyzed for the inclusion of behavior change techniques and also analyzed qualitatively. Two independent coders performed analysis in each phase, and discrepancies were resolved by a third coder.

**Results:**

Substantial agreement was observed between coders (Kappa = 0.63–1.00). ChatGPT-4 provided advice in Traditional Chinese with opening, bullet-point recommendations, and concluding. In phase I, the median Jaccard coefficient was 0.50, indicating moderate consistency. In phase II, specificity was higher for readiness to quit (0.58) and age (0.44) compared to nicotine dependence (0.40). Phase III identified 17 unique behavior change techniques, averaging 10 per response. Qualitative analysis found that ChatGPT-4's advice was somewhat tailored but lacked sufficient detail or contextual appropriateness.

**Conclusions:**

ChatGPT-4 demonstrated moderate consistency, specificity, and inclusion of evidence-based content in its Traditional Chinese cessation advice. Improvements are needed to increase adherence to cessation guidelines and enhance contextual relevance.

**Implications:**

This study is the first to evaluate ChatGPT's ability to deliver smoking cessation advice in Traditional Chinese, a non-dominant language for such large language model. It highlights the feasibility of using generative artificial intelligence for digital health interventions beyond English-speaking settings. The findings demonstrate ChatGPT's potential to support smoking cessation in Chinese-speaking populations and suggest broader applicability for culturally and linguistically underrepresented communities in global tobacco control efforts.

## Introduction

Smoking is a major global public health challenge accounting for over 8 million deaths each year.^1^ While Hong Kong's daily smoking prevalence is among the lowest in the developed world at 9.1% in 2023,^2^ there are still approximately 630 000 cigarette smokers, half of whom will die prematurely due to smoking. Despite the availability of free, evidence-based smoking cessation (SC) services—such as clinics, hotlines, and medications—provided by the Government of the Hong Kong Special Administrative Region of the People's Republic of China, these services are resource-intensive, not easily accessible, and utilized by only about 4% of current smokers.^2^ In contrast, 60% of smokers in Australia^3^ and 34.4% in the United States^4^ reported using these services and medications within past year. This disparity underscores the low utilization of existing cessation services in Hong Kong and highlights the need for innovative, accessible approaches to improve SC support.

The launch of ChatGPT, a large language model (LLM)-based chatbot released by OpenAI in late 2022, marked a significant breakthrough in the landscape of artificial intelligence.^5^ Trained on a vast array of Internet texts, ChatGPT is capable of answering questions, engaging in in-depth conversations, and generating coherent, logically structured texts across a broad spectrum of subjects and languages.^6^ It even passed the United States Medical Licensing Examination, highlighting its potential in medical reasoning and knowledge.^7^ These capabilities are particularly relevant to SC support, which depends on effective communication, empathy, and personalized guidance. SC support is inherently conversational, involving exploration of smokers' motivations and barriers—tasks well aligned with LLMs' strengths in understanding and generating natural language. Moreover, LLMs can provide such support at scale and in real time, offering a practical supplement where access to professional counseling remains limited.

Interest in applying LLMs in health care is soaring. A recent systematic review identified 137 studies evaluating the performance of LLMs providing health advice, among which 135 were evaluating ChatGPT.^8^ Most studies examined clinical topics such as surgery (55 [40.1%]) and medicine (51 [37.2%]), while few focused on primary care (13 [9.5%]). Studies across various fields found that ChatGPT can provide relevant and reasonable advice for health-related questions. For example, one study used ChatGPT to generate responses to 100 health-related questions from five medical specialties.^9^ Patients found these responses more empathetic and useful than those from doctors, while physicians rated them as more accurate than the doctors' responses. Similarly, another study evaluated ChatGPT's responses to 64 alcohol use disorder-related questions and found its responses evidence-based and authentic.^10^

However, how ChatGPT responds to SC queries and whether it can effectively assist smokers to quit remains largely unexplored. As of February 27, 2025, a search of PubMed, Google Scholar, and Web of Science yielded only three studies investigating ChatGPT's performance in tobacco cessation. The first study, involving five experts, concluded that ChatGPT was satisfactory in answering vaping cessation questions.^11^ The second study, based on ratings from seven experts, suggested that while ChatGPT's responses to tobacco-related questions were generally positive, they lacked validity.^12^ However, these conclusions were based on experts' subjective judgments. The third study assessed three ChatGPT chatbots' adherence to SC guidelines and found that most responses were reliable, although the quality of the information varied.^13^

One major limitation of these studies is the exclusive use of English for evaluation queries. Although ChatGPT has primarily been optimized for English, it is capable of communicating in many other languages. Studies focusing exclusively on ChatGPT's performance in English may hinder its potential application for non-English speakers and limit its generalizability to diverse populations. Our literature search did not identify any studies evaluating the performance of ChatGPT in delivering health behavior interventions (eg, SC, physical activity) in Chinese. Nonetheless, limited cross-language evaluations have shown that although ChatGPT performs slightly less effectively in Chinese than in English, it remains capable of generating generally accurate and contextually appropriate health advice in Chinese.^14^^,^^15^

This study assessed the consistency, specificity, and inclusion of behavior change techniques (BCTs)—observable, replicable, and irreducible components of behavioral interventions^16^—in ChatGPT's advice for different types of Traditional Chinese SC queries, the official written language used in Hong Kong. While clinical guidelines suggest including commonly used techniques such as providing information on health risks and benefits, offering social support, and goal setting, systematic mapping of BCTs in Chinese cessation interventions is limited, which further underscores the novelty and significance of evaluating ChatGPT's capability to deliver evidence-based BCTs in this context. We employed GPT-4, the most advanced model available at the start of this study, to ensure the highest level of language understanding and response quality. For consistency, this article will refer to the model as ChatGPT-4 throughout. By assessing ChatGPT-4's ability to provide SC advice in Traditional Chinese, this study aims to explore its potential to support SC efforts in Hong Kong and beyond, extending its applicability for serving culturally and linguistically diverse populations.

## Materials and Methods

### Design

This validation study retrospectively assessed the consistency, specificity, and inclusion of BCTs in ChatGPT-4's SC advice for Traditional Chinese queries, conducted over three phases of evaluation. Three sets of Traditional Chinese SC queries—simple questions, concise vignettes, and detailed vignettes—were used to generate advice between September and November 2023. ChatGPT-4 was accessed through the University of Hong Kong's official service (https://chatgpt.hku.hk/) due to OpenAI's access restrictions in Hong Kong. This service, provided by Azure OpenAI Services through the GPT-4 Application Programming Interface (API), allows users to control several parameters, including ``temperature'' and ``Top P'' (Appendix S1). These parameters influence the randomness of ChatGPT's responses,^17^ with temperature shown to specifically affect output consistency.^18^ While the temperature setting on the ChatGPT website is 0.7,^19^ no consensus exists on the Top P setting. For our evaluation, we used the default values for Azure OpenAI Services, setting the temperature to 0.7 and Top P to 0.95. The temperature value of 0.7 was selected because it reflects the default configuration of the ChatGPT interface, maintaining ecological validity for evaluating real-world user interactions. No additional adjustments or training were applied to ChatGPT-4 for SC. To prevent contamination from prior content, each query was entered into a separate ``new chat'' session.

All prompts used in this study were fully standardized across coders, with identical wording, model parameters, and input format to ensure consistency in ChatGPT-4's input. Prior to data collection, we conducted a small pilot test with several sample inputs to confirm that the prompts were clearly interpreted by the model and produced coherent, contextually appropriate outputs. No major modifications were required following the pilot, and the finalized prompts were then applied consistently across all vignettes.

### Advice Generation and Data Collection

#### Phase I: Assessing Consistency

This phase assessed the consistency of ChatGPT-4's responses to simple SC questions asked at different times. The research team discussed and selected six frequently asked SC questions (Table 1) from a previous randomized controlled trial on instant messaging SC interventions in Hong Kong.^20^ This approach ensured that the selected questions reflected real-world concerns and queries from individuals engaged in the SC process. Although only six questions were selected, each was repeated 10 times to assess the consistency of ChatGPT-4's responses. Two coauthors (XYX and ZJKC), each with more than 2 years of research experience in SC, separately summarized the themes from all advice to generate a coding list. The two researchers' coding lists were then combined and refined to form a final coding list (Appendix S2).

**Table 1:** Consistency of ChatGPT's Responses for Six Most Frequently Asked Smoking Cessation-Related Questions

Question	Jaccard coefficient, median (IQR)	Kappa (95% CI)
I am considering quitting smoking, can you help me?	0.48 (0.39, 0.60)	0.71 (0.58 to 0.84)
I want to quit smoking (*what should I do?*).	0.64 (0.57, 0.71)	0.65 (0.51 to 0.78)
When I feel bored, I tend to smoke (*what should I do?*).	0.50 (0.41, 0.71)	0.70 (0.58 to 0.83)
I haven't been drinking coffee these past few days because I don't want to trigger my craving for cigarettes (*what's your suggestion?*).	0.47 (0.22, 0.69)	0.96 (0.91 to 1.00)
Today is the third day of my nicotine replacement therapy (*what's your suggestion?*).	0.41 (0.26, 0.50)	0.65 (0.52 to 0.79)
I believe I developed the habit of smoking because I live alone (*what should I do?*).	0.37 (0.30, 0.53)	0.72 (0.61 to 0.89)
Overall	0.50 (0.37, 0.64)	0.76 (0.71 to 0.80)

Kappa: 0–0.20: poor agreement, 0.21–0.40: fair agreement, 0.41–0.60: moderate agreement, 0.61–0.80: substantial agreement, 0.81–1.00: almost perfect agreement.

The original questions were in Traditional Chinese and were translated into English here.

The content in parentheses within the question was an implicit query and did not actually appear in the text of the question.

CI = confidence interval.

#### Phase II: Assessing Specificity

This phase assessed whether ChatGPT-4 could tailor SC advice based on specific characteristics in concise vignettes, conducted in October 2023. We created 12 vignettes combining age (adolescent/adult), nicotine dependence (mild/moderate/severe), and readiness to quit (ready/not ready) to test specificity. Each vignette, under 40 words, was input separately into ChatGPT-4, prompted as a SC counselor by entering ``you are a smoking cessation counselor'' and ``Can you help me?'' (Appendix S3) Responses were archived and coded according to the 2008 U.S. Clinical Practice Guideline^21^ and phase I's coding list. This guideline, widely adopted in SC interventions in China^22^ and Hong Kong,^23^ offers a robust framework for effective SC strategies.

The guideline recommends using the 5As (Ask, Advise, Assess, Assist, Arrange) for smokers ready to quit and the 5Rs (Relevance, Risks, Rewards, Roadblocks, Repetition) for those not ready. Since this phase involved single-round conversations, not all 5As or 5Rs were applicable. For example, ChatGPT-4 couldn't ``Ask'' about smoking characteristics or ``Arrange'' follow-up. ``Repetition'' was also not feasible due to the single query. We expected ChatGPT-4 to address the five ``Assist'' components (Quit plan, Medication, Counseling, Social support, and Supplementary material) for ready-to-quit vignettes, and four 5R components (excluding Repetition) for those not ready. The guideline suggests higher-dose nicotine replacement therapy for highly nicotine-dependent smokers, with caution regarding SC medications for adolescents. We expected ChatGPT-4 to recommend higher-dose nicotine replacement therapy for severe dependence and avoid recommending medications for adolescents. The advice was also coded based on phase I's coding list (Appendix S2).

#### Phase III: Assessing Inclusion of BCTs

This phase assessed the inclusion of BCTs in ChatGPT-4's advice for detailed SC vignettes in November 2023. Twenty vignettes (Appendix S4), each containing approximately 150 words on sociodemographic and smoking characteristics, were input into ChatGPT-4 to elicit advice. These vignettes reflected a diverse range of smoker profiles and behaviors. ChatGPT-4 was prompted as a SC counselor with the instructions, ``You are a smoking cessation counselor'' and ``Can you help me?'' ChatGPT-4 was evaluated in its default state without any additional training or prior exposure to the BCT taxonomy, to reflect its typical performance under real-world user conditions. BCTs were identified using the BCTs Taxonomy for SC,^24^ which includes 43 techniques grouped into four functions: (1) improving motivation, (2) enhancing capacity, (3) facilitating adjuvant activities (eg, SC medications), and (4) supporting other BCTs (eg, building rapport). Since this phase involved only one-round conversations with no physical contact, 17 BCTs related to interactive communication and physical measurements were not applicable. These included, for example, RI1 (Assess current and past smoking behavior) and BM1 (Measure CO). After screening, nine BCTs were excluded for simplifying the analysis, leaving 17 BCTs for further examination (adapted BCT checklist in Appendix S5).

### Coding Procedure

In phases I and II, two coauthors (XYX and ZJKC) independently coded the ChatGPT-4 advice according to the corresponding label lists. Discrepancies were addressed by a third coauthor (MJZ). All coders discussed and reached consensus on the label meanings and coding procedures before proceeding with the coding.

In phase III, one coauthor (XYX), who had prior expertise in the BCTs Taxonomy for behavioral support in SC, developed a coding guideline to familiarize the team with the taxonomy and provide examples of how to code ChatGPT-4's advice using the taxonomy (Appendix S6). One coauthor (YTDC) and a research nurse (not listed as an author), who had over 10 years of SC counseling experience, followed the coding guideline to identify BCTs in each piece of ChatGPT-4 advice. Discrepancies were addressed by coauthor XYX. The two coders also provided qualitative feedback on ChatGPT-4's performance based on their expertise.

### Statistical Analysis

In all phases, Kappa coefficients were calculated to determine the agreement between the two coders by comparing the observed proportion of agreement to the expected proportion of agreement by chance. A Kappa value greater than 0.60 indicates substantial agreement.^25^

In phase I, the themes from the coding list that appeared in each piece of advice were coded to represent the content of the advice. To determine content consistency between different pieces of advice for the same SC question, Jaccard coefficients were calculated for each pair of pieces of advice. The Jaccard coefficient is an algorithm that can be used to calculate text similarity.^26^ It is defined as the size of the intersection divided by the size of the union of the sets, mathematically expressed as J(A, B) = |A ∩ B| / |A ∪ B|. This coefficient ranges from 0 to 1, where a higher value indicates greater similarity between the sets. In phase I, the primary outcome for measuring consistency of ChatGPT-4's SC advice was Jaccard coefficient.

In phase II, advice was grouped based on different conditions of a factor in the vignettes (eg, the ready-to-quit group and the not-ready-to-quit group). The number of pieces of advice that aligned with the 2008 U.S. Clinical Practice Guideline in each group was then compared. Additionally, pairwise comparisons of themes from the phase I label list of advice were made for vignettes that varied by one factor but were identical in the other two factors. This was done to determine the variability of the advice attributed to that specific factor. Jaccard distance, the complement of the Jaccard coefficient, calculated as D(A, B) = 1–J(A, B), was used to indicate variability. In phase II, the primary outcomes for measuring the specificity of ChatGPT-4's SC advice were the number of pieces of advice that complied with the guideline in different groups and the Jaccard distance. The Jaccard distances attributed to these three factors were compared using Wilcoxon rank-sum tests because they were not normally distributed.

In phase III, the primary outcome for measuring the inclusion of BCTs of ChatGPT-4's SC advice was the occurrence frequency of each BCT. Qualitative feedbacks for these advice from two coders were summarized and presented.

## Results

In all three phases, ChatGPT-4 provided advice in Traditional Chinese. Most of its advice (33 out of 60 in phase I, 11 out of 12 in phase II, and 19 out of 20 in phase III) followed a consistent pattern: an introductory or greeting paragraph, several bullet points of recommendations, and a concluding summary paragraph.

### Phase I: Assessing Consistency

Table 1 shows that the Kappa statistics for coding each set of ChatGPT-4's advice for the six simple SC questions ranged from 0.65 to 0.96, indicating substantial to almost perfect agreement between the coders in phase I. The overall median (Interquartile Range [IQR]) Jaccard coefficient for any two pieces of advice for the same question was 0.50 (0.37, 0.64), suggesting a moderate level of content consistency.

### Phase II: Assessing Specificity

Table 2 shows that the Kappa statistics ranged from 0.63 to 1.00 for coding using the guideline. The Kappa statistic for coding with the phase I label list was 0.75 (95% CI = 0.69 to 0.82), indicating substantial to almost perfect agreement between the coders in phase II.

**Table 2:** Compliance With 2008 U.S. Clinical Practice Guideline of Advice to Concise Vignettes

Rating criteria	Number of pieces of advice complied with the recommended approach			Kappa (95% CI)
Assist	Ready-to-quit (*N* = 6)	Not-ready-to-quit (*N* = 6)		0.63 (0.40 to 0.85)
A1 Quit plan	6	6		
A2 Medication recommendation	6	6		
A3 Practical counseling	6	6		
A4 Intra-treatment social support	6	6		
A5 Supplementary material	0	0		
Rs	Ready-to-quit (*N* = 6)	Not-ready-to-quit (*N* = 6)		0.64 (0.41 to 0.86)
R1 Relevance	1	2		
R2 Risks	0	1		
R3 Rewards	2	4		
R4 Roadblocks	6	6		
Cautions against prescription of SC medications to adolescents	Adolescents (*N* = 6)	Adults (*N* = 6)		1.00 (1.00 to 1.00)
	0	0		
Recommendation of higher-dose preparations of NRT or NRT combinations	Mild nicotine dependence (*N* = 4)	Moderate nicotine dependence (*N* = 4)	Severe nicotine dependence (*N* = 4)	1.00 (1.00 to 1.00)
	0	0	0	

Subsections of the **Assist** strategy were used as other four A strategies (ask, advise, assess, and arrange) were not applicable in this analysis. Quit plan refers to assisting the patient with a quit plan; medication recommendation involves suggesting approved medications; practical counseling means delivering problem-solving or skills training; intra-treatment social support is about providing a supportive clinical environment; supplementary material offers information on other smoking cessation services like quitlines.

**Repetition** strategy from 5Rs is not included in this table since this is a one-time advice. Relevance refers to provide personally relevant motivational information; risks refers to potential negative consequences of tobacco use; rewards refers to potential benefits of stopping tobacco use; roadblocks refers to identify barriers or impediments to quitting and provide treatment that could address barriers.

CI = confidence interval; NRT = nicotine replacement therapy; SC = smoking cessation.

As shown in Table 2, ChatGPT-4's advice for all 12 concise SC vignettes included recommendations for setting a quit date (Quit plan), using SC medications (Medication recommendation), providing problem-solving (Practical counseling), and maintaining a supportive tone (intra-treatment social support). However, it did not include information on other SC services (Supplementary material). All advice included these four components of Assist, regardless of readiness to quit.

For the six not-ready-to-quit vignettes, ChatGPT-4 provided motivational information (Relevance) for two, negative consequences (Risks) for one, benefits of quitting (Rewards) for four, and discussed barriers and strategies (Roadblocks) for all six. Fewer ready-to-quit vignettes followed the 4Rs principle, but the differences were not statistically significant. ChatGPT-4 recommended SC medications for all vignettes but did not suggest higher-dose nicotine replacement therapy or nicotine replacement therapy combinations for severe dependence or caution against prescribing SC medications for adolescents.

Table 3 shows that, when examined in terms of the phase I label list, Jaccard distances attributed to age (Median [IQR]: 0.44 [0.36, 0.51]) and quitting readiness (0.58 [0.51, 0.69]) were significantly higher than that for nicotine dependence (0.40 (0.32, 0.44)), *p*_(age)_ = .03, *p*_(readiness to quit)_ = .02. This suggests greater variation in advice due to different age groups and quitting readiness than due to nicotine dependence.

**Table 3:** Jaccard Distances Attributed to Differential Factors in Terms of Phase I Label List

Differential factors	Jaccard distance, median (IQR)	*p* value^a^
Nicotine dependence [Reference]	0.40 (0.32, 0.44)	NA
Age	0.44 (0.36, 0.51)	.03^*^
Readiness	0.58 (0.51, 0.69)	.02^*^

a *p* values from Wilcoxon rank-sum tests. * < 0.05

### Phase III: Assessing Inclusion of BCTs

**Figure 1:**
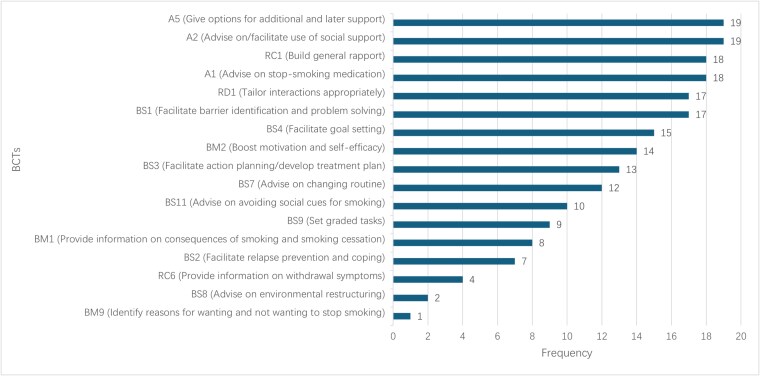
BCTs occurrence frequency within ChatGPT's advice for the 20 detailed SC vignettes. BCT = behavior change technique; SC = smoking cessation.

The Kappa statistic for phase III coding was 0.68 (95% CI = 0.60 to 0.76), indicating substantial agreement between coders. As shown in Figure 1, ChatGPT-4 included 17 unique BCTs in its advice for 20 detailed SC vignettes, with an average of 10 BCTs per piece of advice. The most frequently used BCTs were BS1 (barrier identification and problem solving [17/20]), A1 (advise on stop-smoking medication [18/20]), A2 (advise on/facilitate social support [19/20]), A5 (additional support options [19/20]), RD1 (tailor interactions [17/20]), and RC1 (build rapport [18/20]). Table 4 shows examples of advice for each BCT.

**Table 4:** Examples of BCTs Included in ChatGPT's Advice

BCTs	Excerpts of ChatGPT's advice^a^
**BM1** Provide information on consequences of smoking and smoking cessation	Understanding the benefits of quitting smoking is very important for your physical health, family, and financial situation (The specific benefits of these three aspects were mentioned by ChatGPT in the original advice but are omitted here for brevity).
**BM2** Boost motivation and self-efficacy	You've made the decision to change your life, believe in yourself that you can successfully quit smoking!
**BM9** Identify reasons for wanting and not wanting to stop smoking	Identify the reasons to quit smoking: Write these reasons down and remind yourself when you need motivation.
**BS1** Facilitate barrier identification and problem solving	Change lifestyle habits: *Try to identify the triggers that cause you to smoke*,^b^ such as stress, boredom, or being with certain friends. Then, try to change these habits to reduce the temptation to smoke.
**BS2** Facilitate relapse prevention and coping	Don't be discouraged: There may be relapses during the process of quitting smoking. Don't be discouraged. Continue to persist in quitting and learn from it on how to avoid relapsing again.
**BS3** Facilitate action planning/develop treatment plan	Make a plan to quit smoking: *First, create a plan to quit smoking*. Determine your quit goals and dates, and announce your decision to family and friends.
**BS4** Facilitate goal setting	Make a plan to quit smoking: First, create a plan to quit smoking. *Determine your quit goals and dates*, and announce your decision to family and friends.
**BS7** Advise on changing routine	*Change lifestyle habits*: Try to identify the triggers that cause you to smoke, such as stress, boredom, or being with certain friends. Then, try to change these habits to reduce the temptation to smoke.
**BS8** Advise on environmental restructuring	Stay away from temptations: Avoid situations where you're with smokers, and *clean out all tobacco products from your home and car*.
**BS9** Set graded tasks	Try to reduce the number of times you smoke: You can try to gradually reduce the number of cigarettes you smoke each day, thereby reducing the harmful impact on your health. For example, you could try to reduce your daily consumption from 15 cigarettes to 10.
**BS11** Advise on avoiding social cues for smoking	Stay away from temptations: *Avoid situations where you're with smokers*, and clean out all tobacco products from your home and car.
**A1** Advise on stop-smoking medication	Use nicotine substitutes: Nicotine replacement therapies, such as nicotine patches, gum, or inhalers containing nicotine, can help alleviate nicotine cravings when quitting smoking.
**A2** Advise on/facilitate use of social support	Make a plan to quit smoking: First, create a plan to quit smoking. Determine your quit goals and dates, and *announce your decision to family and friends*.
**A5** Give options for additional and later support	Seek professional help: Smoking cessation counseling, professional doctors, or pharmacological treatments can all increase the chances of successfully quitting smoking.
**RD1** Tailor interactions appropriately	I understand your concerns. Quitting smoking is beneficial for both your and your family's health, especially considering your son's asthma. (Child with asthma was mentioned in the vignette)
**RC1** Build general rapport	I hope these suggestions are helpful to you. Best of luck with your quit smoking journey!
**RC6** Provide information on withdrawal symptoms	Understand withdrawal symptoms: During the process of quitting smoking, you may experience some withdrawal symptoms such as anxiety, irritability, and increased appetite. Understanding these symptoms can help you better cope with them.

a The original advice was in Traditional Chinese and was translated into English here.

b Each advice could include more than one BCT. Sections related to the specific BCT have been italicized.

BCT = behavior change technique.

**Figure 2:**
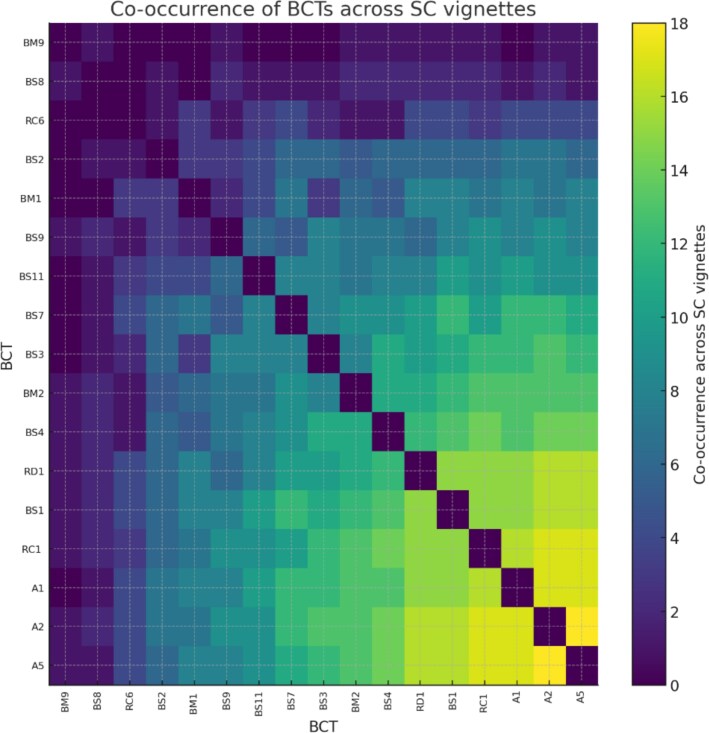
BCT cooccurrence across 20 smoking cessation vignettes. BCT = behavior change technique; SC = smoking cessation.

Figure 2 shows that across 20 SC vignettes, ChatGPT most frequently combined social support (A2, A5) and pharmacological advice (A1) techniques. The strongest cooccurrence was between A2 and A5, followed by frequent pairing of A1 with A2 and A5. Moderate clustering occurred among BS1, BS3, and BS4, reflecting integration of planning and problem-solving elements. In contrast, BM9, BS8, and RC6 rarely co-occurred with other techniques, suggesting limited focus on motivational reflection and environmental strategies.

Table 5 highlights three themes from the qualitative analysis: specificity, inappropriateness, and vagueness. ChatGPT-4 tailored its advice based on key vignette information, such as recommending SC resources for low-income smokers and emphasizing quitting benefits for a smoker with a child suffering from asthma. However, it provided inappropriate information for one vignette by referencing Taiwan-based services for a Hong Kong scenario and made confusing statements like ``I am not a smoking cessation counselor, but an AI assistant.'' Additionally, it gave general advice, such as ``quitting smoking is good for your health'', and vague suggestions, like suggesting setting a quit date without providing specific instructions.

**Table 5:** Qualitative Analysis on ChatGPT's Advice

Theme	Excerpt of ChatGPT's advice^a^	Comments
Specificity	I understand your concerns. Quitting smoking is beneficial for both your and your family's health, especially considering your son's asthma (Child with asthma was mentioned in the vignette).	It mentioned one of the most important motivations for the smoker to quit.
	Understanding the benefits of quitting smoking is very important for your physical health, family, and financial situation (The specific benefits of these three aspects were mentioned by ChatGPT in the original advice but are omitted here for brevity).	It mentioned the most important motivations for the smoker to quit.
	Seek free or low-cost smoking cessation resources: Many cities and medical organizations offer free or low-cost smoking cessation programs and counseling. (Low income was mentioned in the vignette).	It considered the smoker's economic situation and needs, and introduced free smoking cessation resources.
Inappropriateness	In Taiwan, you can call the free smoking cessation hotline: 0800-63-63-63, to get professional advice and support.	It should give information of smoking cessation resources in Hong Kong.
	I am not a smoking cessation counselor, but an AI assistant. However, I can provide you with information about quitting smoking and insomnia.	It is confusing to see a statement that ``I am not a smoking cessation counselor''.
Vagueness	Quitting smoking is a great decision that benefits both your health and your family significantly.	It should give more precise and personalized information rather than simply mention benefits.
	List the reasons for quitting smoking to remind yourself during the process of quitting (Being female was mentioned in the input).	It should mention specifically female-concerning problem such as dry skin and beauty damage.
	Set a definite date to quit smoking and reduce the amount you smoke before then.	It should support user to choose a special date to quit such as birthday or wedding anniversary.
	You can also join a smoking cessation group to share experiences and receive support from other people who are quitting smoking.	It should provide more information of smoking cessation services which facilitate smoking cessation groups

a The original advice was in Traditional Chinese and was translated into English here.

## Discussion

This first study to assess advice delivered by an LLM for SC queries in Traditional Chinese showed that ChatGPT-4 does not provide identical advice for a question asked at different times. It tailors its advice to specific demographic and smoking characteristics and incorporates various BCTs when addressing SC queries.

In terms of consistency, phase I showed that the median Jaccard coefficient between two pieces of advice for the same question was around 0.5, indicating approximately 50% overlap in ChatGPT-4's responses when the same question was asked at different times. This variability is consistent with findings from Shin and Ramanathan,^27^ who reported that the content of ChatGPT-4's responses can differ across instances of the same prompt. Such variability suggests that ChatGPT-4 generates responses with varied focus, rather than repeating the same answer, which may be less appealing, as seen with rule-based SC chatbots.^28^ As a generative model, ChatGPT generates each response dynamically rather than retrieving preset texts; thus, some variability is expected. When such variability remains relevant and evidence-based, it might enhance engagement and personalization, though excessive variation may compromise safety and fidelity to guidelines.

In terms of specificity, phase II showed that ChatGPT-4 was sensitive to a smoker's age and readiness to quit, responding more differently based on these factors than on nicotine dependence. Phase III qualitative results also showed that ChatGPT-4 was attentive to smokers' economic status and specific motivations to quit, providing tailored advice. This result aligns with the high relevance of ChatGPT-4's response to inputted vaping cessation-related questions reported by Samia et al.,^11^ which, together with our results, highlights ChatGPT-4's capability to personalize SC advice. However, the lack of tailoring to nicotine dependence remains an important limitation, as it may affect the appropriateness of pharmacotherapy recommendations and the overall clinical safety of the advice.

In terms of the inclusion of BCTs, phase III showed that ChatGPT-4 demonstrated the ability to incorporate a broad range of BCTs into its SC advice, including 17 unique BCTs with an average of 10 per response. The most frequently used techniques involved social support (A2, A5), pharmacotherapy advice (A1), and barrier identification and problem solving (BS1), often co-occurring within the same response. These combinations suggest that ChatGPT-4 can deliver multi-component behavioral strategies resembling those commonly used in human-delivered counseling, emphasizing interpersonal support, problem solving, and medication use. However, several evidence-based BCTs were underrepresented, such as relapse prevention planning and environmental restructuring. Although no single intervention would necessarily employ all BCTs, these gaps likely reflect constraints in ChatGPT's training data or generative focus, which tend to prioritize motivational and informational content over structured behavioral maintenance strategies. Addressing these limitations—through explicit prompt engineering or fine-tuning—could enhance the model's ability to deliver more comprehensive, guideline-aligned cessation counseling.

Consistent with findings from other studies on ChatGPT,^13^^,^^27^^,^^29^ we observed that ChatGPT-4 occasionally provided inappropriate information in its SC advice. For instance, in response to a query, ChatGPT-4 provided information about cessation services in Taiwan while it was from Hong Kong. This likely occurred because the query was in Traditional Chinese, but Hong Kong was not specified, leading ChatGPT-4 to provide information from another region that uses Traditional Chinese. Although such errors may appear minor, they could mislead users seeking professional help, reduce trust in digital health tools, and pose potential safety risks if users act on inaccurate information. These findings underscore the importance of regional customization, human oversight, and safety mechanisms when deploying LLM-based intervention in real-world settings.

Additionally, ChatGPT-4 sometimes offered general statements and vague suggestions. Such advice may be perceived as irrelevant and unhelpful, potentially reducing engagement and limiting its effectiveness in SC. Furthermore, in phase II, ChatGPT-4's advice did not fully align with the 2008 U.S. Clinical Practice Guidelines. This aligns with recent study, which found that only 57.1% of ChatGPT chatbot responses adhered to cessation guidelines,^13^ indicating that ChatGPT-4's SC advice would benefit from closer alignment with established guidelines. In particular, its lack of tailoring to nicotine dependence represents a clinically important gap, as dependence level determines the appropriateness of pharmacotherapy and the intensity of behavioral support. Enhancing ChatGPT's ability to recognize and respond to nicotine dependence could therefore improve both safety and treatment personalization.

In this study, queries were made in Traditional Chinese, and ChatGPT-4 responded appropriately in the same language, suggesting that ChatGPT-4 can effectively provide relevant SC advice in a non-dominant language. Beyond the local context, this study carries broader global significance. It demonstrates ChatGPT-4's potential to deliver culturally and linguistically relevant health advice in a non-English setting, addressing an important gap in digital health research that has predominantly focused on English-language users. These findings highlight the feasibility of extending LLM-based behavioral interventions to linguistically diverse and underrepresented populations worldwide.

As suggested by recent studies,^27^^,^^30^^,^^31^ designing and refining input prompts (prompt engineering) may guide ChatGPT to produce more accurate, relevant, and contextually appropriate advice. Future prompt engineering efforts to enhance ChatGPT's performance in SC should consider incorporating information about local cessation resources, strengthening adherence to authoritative clinical guidelines and behavioral strategies, and encouraging more specific and actionable recommendations. In addition, prompt designs could explicitly instruct the model to tailor advice based on key smoker characteristics—such as nicotine dependence level—to improve both safety and personalization.

Several limitations should be noted. First, only one round of interaction with ChatGPT-4 was conducted. While this design enabled a standardized evaluation across vignettes, longer or iterative conversations might allow the model to gather more contextual information and demonstrate greater counseling capacity over time. Second, the temperature was fixed at 0.7, which was selected to reflect the default configuration of the ChatGPT but may influence response variability; different settings may lead to different levels of consistency. Third, the identification of BCTs relied on human coding, which, although based on a standardized taxonomy and double-coding process, may still involve subjective interpretation. Fourth, the relatively small sample size limited our ability to infer ChatGPT-4's performance in real-world settings. Lastly, we only analyzed responses to SC queries in Traditional Chinese, which may limit the generalizability of these findings to other languages and cultural contexts.

In conclusion, without additional adjustment or training, ChatGPT-4 provided relevant and varied advice, tailored to specific characteristics of smokers, and incorporated BCTs effective for SC in its responses to SC queries in Traditional Chinese. However, it occasionally provided inappropriate or overly general information, offered vague suggestions, and did not fully align with the established guideline. While ChatGPT-4 shows potential for delivering general SC advice in Traditional Chinese, it could be further improved by addressing these limitations.

## Supplementary Material

Supplementary_Material_1_ntaf267(1)

Supplementary_Material_2_ntaf267(1)

Supplementary_Material_3_ntaf267(1)

Supplementary_Material_4_ntaf267(1)

Supplementary_Material_5_ntaf267(1)

Supplementary_Material_6_ntaf267(1)

## Data Availability

All prompts used to query ChatGPT-4 and coding materials are available in the appendix.
