# Supplementary figures and images for: ChatGPT-4's Consistency, Specificity, and Inclusion of Behavior Change Techniques in Delivering Smoking Cessation Advice in Traditional Chinese: A Content Analysis
Source: Nicotine Tob Res. 2025 Dec 24;28(6):1006–15. doi: 10.1093/ntr/ntaf267 (PMC13196701; doi:10.1093/ntr/ntaf267)

# Supplementary Material 1. Screenshot of ChatGPT service provided by HKU


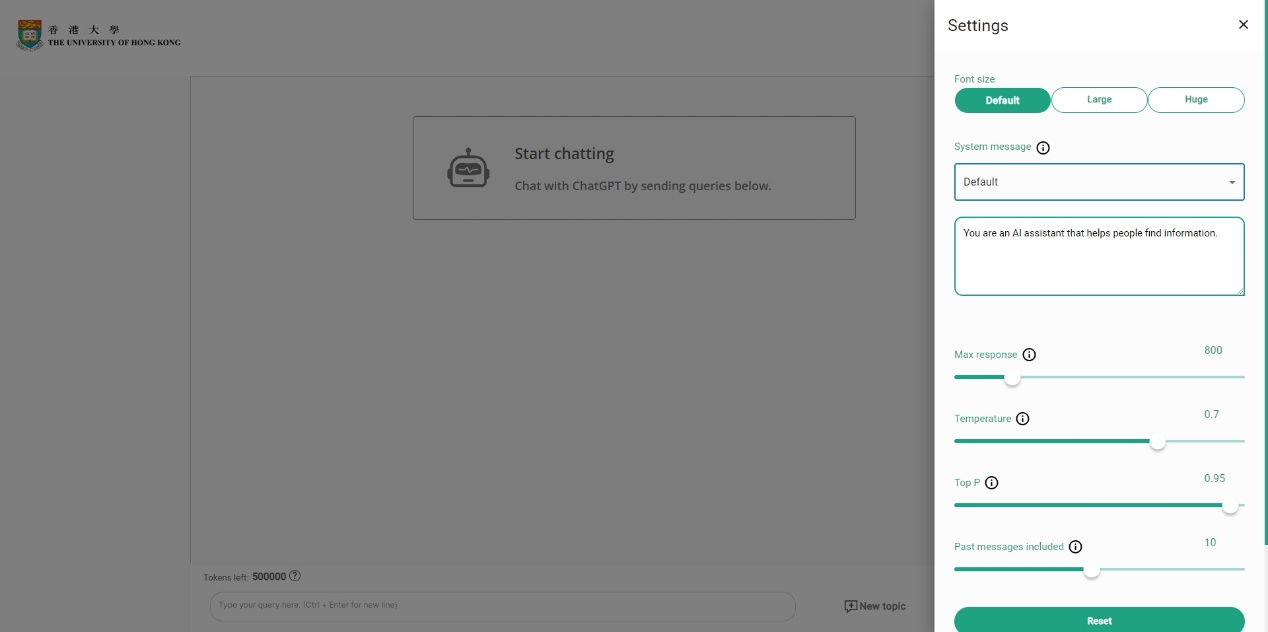

Supplement: Supplementary_Material_1_ntaf267(1) [file supplementary_material_1_ntaf267(1).docx]
