# Supplementary material for: ChatGPT-4's Consistency, Specificity, and Inclusion of Behavior Change Techniques in Delivering Smoking Cessation Advice in Traditional Chinese: A Content Analysis
Source: Nicotine Tob Res. 2025 Dec 24;28(6):1006–15. doi: 10.1093/ntr/ntaf267 (PMC13196701; doi:10.1093/ntr/ntaf267)
Supplement: Supplementary_Material_2_ntaf267(1) [file supplementary_material_2_ntaf267(1).docx]

# Supplementary Material 2. Phase 1 content labels

| Label number | Label |
| --- | --- |
| A1^*^ | Recommend to seek supportive groups or people |
| A2^*^ | Advise to set up a quit date |
| A3^*^ | Advise to seek professional assistance |
| A4^*^ | Advise to avoid smoking cues (e.g alcohol, stress) |
| A5^*^ | Advise to develop healthy habits (e.g running and reading), keep busy or divert attention from smoking |
| A6^*^ | Encourage to seek NRT ^a^ or smoking cessation (SC) medications |
| A7^*^ | Advise to seek and determine the motivation to quit smoking |
| A8^*^ | Encourage to continue to quit smoking after relapse |
| A9^*^ | Encourage to seek substitutes, expect NRT and SC medications (e.g candy or gum) |
| A10^*^ | Advise to prepare for the difficulties may encounter during quitting |
| A11^*^ | Advise to set up a quit plan |
| A12^*^ | Affirmative encouragement |
| A13^*^ | Advise to learn to cope with stress or negative emotion without smoking |
| A14^*^ | Advise to reward oneself contingent on effort or progress |
| A15^*^ | Advise to change habit and routine |
| A16^*^ | Encourage to maintain patient and positive |
| A17^*^ | Advise to exercise |
| A18^*^ | Encourage to have deep breathing or meditation |
| B1 | Recommend to seek supportive groups or people |
| B2 | Advise to set up a quit date |
| B3 | Advise to seek professional assistance |
| B4 | Advise to avoid smoking cues (e.g alcohol, stress) |
| B5 | Advise to develop healthy habits (e.g running and reading), keep busy or divert attention from smoking |
| B6 | Encourage to seek NRT or SC medications |
| B7 | Advise to seek and determine the motivation to quit smoking |
| B8 | Encourage to continue to quit smoking after relapse |
| B9 | Encourage to seek substitutes, expect NRT and SC medications (e.g candy or gum) |
| B10 | Advise to prepare for the difficulties may encounter during quitting |
| B11 | Advise to set up a quit plan |
| B12 | Affirmative encouragement |
| B13 | Advise to learn to cope with stress or negative emotion without smoking |
| B14 | Advise to reward oneself contingent on effort or progress |
| B15 | Advise to change habit and routine |
| B16 | Encourage to maintain patient and positive |
| B17 | Advise to exercise |
| B18 | Encourage to have deep breathing or meditation |
| C1 | Recommend to seek supportive groups or people |
| C2^*^ | Encourage to develop a new hobby during leisure time (e.g read books or do sports) |
| C3 | Encourage to have deep breathing or meditation |
| C4 | Advise to seek professional assistance |
| C5 | Encourage to seek substitutes, expect NRT and SC medications (e.g candy or gum) |
| C6 | Encourage to seek NRT or SC medications |
| C7 | Advise to exercise |
| C8 | Advise to develop healthy habits (e.g running and reading), keep busy or divert attention from smoking |
| C9^*^ | Advise to interact with family or friends |
| C10^*^ | Warn about the harms of smoking or remind about the benefits of quitting |
| C11 | Advise to reward oneself contingent on effort or progress |
| C12^*^ | Advise to use smoking cessation apps to assist quitting |
| D1^*^ | Recommend to drink more water or juice to replace coffee |
| D2 | Encourage to have deep breathing or meditation |
| D3 | Advise to seek professional assistance |
| D4^*^ | Encourage to maintain enough sleep time |
| D5 | Recommend to seek supportive groups or people |
| D6 | Advise to develop healthy habits (e.g running and reading), keep busy or divert attention from smoking |
| D7 | Advise to interact with family or friends |
| D8^*^ | Advise to document quitting progress |
| D9 | Advise to reward oneself contingent on effort or progress |
| D11 | Advise to exercise |
| D12 | Encourage to seek substitutes, expect NRT and SC medications (e.g candy or gum) |
| E1^*^ | List out the advantages of having NRT (e.g relieve some of the withdrawal symptoms) |
| E2 | Encourage to have deep breathing or meditation |
| E3^*^ | Suggest appropriate usage of NRT |
| E4 | Recommend to seek supportive groups or people |
| E5 | Advise to develop healthy habits (e.g running and reading), keep busy or divert attention from smoking |
| E6 | Advise to exercise |
| E7 | Advise to interact with family or friends |
| E8 | Encourage to maintain patient and positive |
| E9^*^ | Give praise for the effort and progress the smoker is making |
| E10^*^ | Encourage to maintain motivation to quit smoking |
| E11 | Encourage to seek substitutes, expect NRT and SC medications (e.g candy or gum) |
| E12 | Advise to seek professional assistance |
| F1 | Advise to develop healthy habits (e.g running and reading), keep busy or divert attention from smoking |
| F2 | Advise to seek professional assistance |
| F3 | Advise to avoid smoking cues (e.g alcohol, stress) |
| F4 | Encourage to have deep breathing or meditation |
| F5 | Advise to set up a quit date |
| F6 | Encourage to seek substitutes, expect NRT and SC medications (e.g candy or gum) |
| F7 | Encourage to seek NRT or SC medications |
| F8 | Advise to exercise |
| F9^*^ | Advise to make new friends |
| F10^*^ | Suggest to attend more social events |
| F11 | Recommend to seek supportive groups or people |
| F12 | Advise to reward oneself contingent on effort or progress |
| F13^*^ | Suggest to pay attention to health |

*Note*: The A, B, ..., F in Label number corresponds to Q1, Q2, ..., Q6 respectively. There are some overlaps among these 6 sets of content labels, with a total of 32 distinctive different labels (indicated with ^*^).

^a^ NRT=Nicotine replacement therapy
