# Supplementary material for: ChatGPT-4's Consistency, Specificity, and Inclusion of Behavior Change Techniques in Delivering Smoking Cessation Advice in Traditional Chinese: A Content Analysis
Source: Nicotine Tob Res. 2025 Dec 24;28(6):1006–15. doi: 10.1093/ntr/ntaf267 (PMC13196701; doi:10.1093/ntr/ntaf267)
Supplement: Supplementary_Material_3_ntaf267(1) [file supplementary_material_3_ntaf267(1).docx]

# Supplementary Material 3. Phase 2 vignettes

**Table 1. Summary of the 12 vignettes in Phase 2**

| vignette | Age | Nicotine dependence level | Ready to quit |
| --- | --- | --- | --- |
| 1 | Adolescent | Low | Yes |
| 2 | Adolescent | Low | No |
| 3 | Adolescent | Middle | Yes |
| 4 | Adolescent | Middle | No |
| 5 | Adolescent | High | Yes |
| 6 | Adolescent | High | No |
| 7 | Adult | Low | Yes |
| 8 | Adult | Low | No |
| 9 | Adult | Middle | Yes |
| 10 | Adult | Middle | No |
| 11 | Adult | High | Yes |
| 12 | Adult | High | No |

**Table 2. Specific details of the Phase 2 vignettes**

| Number | Vignette ^a^ |
| --- | --- |
| 1 | **You are a smoking cessation counselor**. I am fifteen years old, and I smoke my first cigarette an hour after I wake up. On average, I smoke five cigarettes a day. I hope to try quitting smoking. **Can you help me**? |
| 2 | **You are a smoking cessation counselor**. I am fifteen years old, and I smoke my first cigarette an hour after I wake up. On average, I smoke five cigarettes a day, but I do not want to quit smoking. **Can you help me**? |
| 3 | **You are a smoking cessation counselor**. I am fifteen years old, and I need to smoke my first cigarette within thirty minutes of waking up. On average, I smoke fifteen cigarettes a day. I hope to try quitting smoking. **Can you help me**? |
| 4 | **You are a smoking cessation counselor**. I am fifteen years old, and I need to smoke my first cigarette within thirty minutes of waking up. On average, I smoke fifteen cigarettes a day, but I do not want to quit smoking. **Can you help me**? |
| 5 | **You are a smoking cessation counselor**. I am fifteen years old, and I need to smoke my first cigarette within five minutes of waking up. On average, I smoke twenty-five cigarettes a day, and I hope to try quitting smoking. **Can you help me**? |
| 6 | **You are a smoking cessation counselor**. I am fifteen years old, and I need to smoke my first cigarette within five minutes of waking up. On average, I smoke twenty-five cigarettes a day, but I do not want to quit smoking. **Can you help me**? |
| 7 | **You are a smoking cessation counselor**. I am forty-five years old, and I smoke my first cigarette an hour after I wake up. On average, I smoke five cigarettes a day. I hope to try quitting smoking. **Can you help me**? |
| 8 | **You are a smoking cessation counselor**. I am forty-five years old, and I smoke my first cigarette an hour after I wake up. On average, I smoke five cigarettes a day, but I do not want to quit smoking. **Can you help me**? |
| 9 | **You are a smoking cessation counselor**. I am forty-five years old, and I need to smoke my first cigarette within thirty minutes of waking up. On average, I smoke fifteen cigarettes a day. I hope to try quitting smoking. **Can you help me**? |
| 10 | **You are a smoking cessation counselor**. I am forty-five years old, and I need to smoke my first cigarette within thirty minutes of waking up. On average, I smoke fifteen cigarettes a day, but I do not want to quit smoking. **Can you help me**? |
| 11 | **You are a smoking cessation counselor**. I am forty-five years old, and I need to smoke my first cigarette within five minutes of waking up. On average, I smoke twenty-five cigarettes a day, and I hope to try quitting smoking. **Can you help me**? |
| 12 | **You are a smoking cessation counselor**. I am forty-five years old, and I need to smoke my first cigarette within five minutes of waking up. On average, I smoke twenty-five cigarettes a day, but I do not want to quit smoking. **Can you help me**? |

^a^ The original vignettes were in Traditional Chinese and were translated into English here.
