# Supplementary material for: ChatGPT-4's Consistency, Specificity, and Inclusion of Behavior Change Techniques in Delivering Smoking Cessation Advice in Traditional Chinese: A Content Analysis
Source: Nicotine Tob Res. 2025 Dec 24;28(6):1006–15. doi: 10.1093/ntr/ntaf267 (PMC13196701; doi:10.1093/ntr/ntaf267)
Supplement: Supplementary_Material_4_ntaf267(1) [file supplementary_material_4_ntaf267(1).docx]

# Supplementary Material 4. Phase 3 vignettes and BCTs checklist

**Table 1. Summary of the 20 vignettes in Phase 3**

| Vignette | Age | Sex | Problem focused |
| --- | --- | --- | --- |
| 1 | Middle-aged | Male | Withdrawal symptoms and COPD |
| 2 | Middle-aged | Female | Family health and weight gain after quitting |
| 3 | Middle-aged | Male | Social cues |
| 4 | Middle-aged | Male | High blood pressure |
| 5 | Middle-aged | Female | Single mother and pressure coping |
| 6 | Middle-aged | Male | Low-income status |
| 7 | Middle-aged | Female | Pregnancy |
| 8 | Middle-aged | Female | Type 2 Diabetes Mellitus |
| 9 | Young adult | Male | Mental health problem |
| 10 | Older adult | Male | Post-stroke |
| 11 | Older adult | Female | Liver problem |
| 12 | Young adult | Female | Fracture |
| 13 | Older adult | Male | Chronic bronchitis |
| 14 | Teenagers | Male | Insomnia |
| 15 | Young adult | Female | Chronic gastritis |
| 16 | Middle-aged | Male | Hyperlipidemia |
| 17 | Middle-aged | Male | Cancer risks |
| 18 | Teenagers | Female | Habits |
| 19 | Older adult | Male | Social factors |
| 20 | Older adult | Female | Respiratory disease |

**Table 2. Specific details of the Phase 3 vignettes**

| Number | Vignette ^a^ |
| --- | --- |
| 1 | **You are a smoking cessation counselor**. I am forty-eight years old, living with a four-year-old daughter and my wife. I have been smoking for twenty-five years. Recently, I have found myself prone to breathlessness, frequent coughing, and producing yellow, thick phlegm. I have been preliminarily diagnosed with chronic obstructive pulmonary disease (COPD). Due to the increase in tobacco taxes and my friends attempting to quit, I wish to try quitting smoking. My wife also hopes that I can successfully quit. Previously, I smoked an average of twenty cigarettes a day, gradually reducing to six to seven cigarettes a day. I hope I can completely quit smoking within a week. I need to smoke my first cigarette four hours after waking up. A week ago, I attempted to start quitting, but I felt anxious and had headache, so I smoked again on the sixth day. I am not familiar with nicotine replacement therapy, only knowing from friends that those products are quite expensive. Usually, I smoke with my colleagues. Due to being busy with work, I do not have time to exercise. **Can you help me**? |
| 2 | **You are a smoking cessation counselor**. I am fifty-six years old. Currently, I am a homemaker living with my non-smoking husband, son, and grandmother. I do not have any long-term illnesses. I need to smoke my first cigarette two hours after waking up. My husband hopes I will quit smoking again for the health of our son, who has severe asthma. I have tried to quit smoking but cannot last more than three days due to severe anxiety. Currently, I smoke only a few cigarettes, about an average of five a day. I do not have the intention to quit again; I feel it's not a problem as long as I do not smoke near my son. There was a time when the pressure from people around me to quit smoking was so great that I ended up smoking more than twenty cigarettes in one day. I do not have the habit of exercising. I am of normal weight but worry that quitting smoking will lead to weight gain. Although I do not currently intend to quit, my husband is very supportive of me quitting. However, he does not know how to encourage me to quit. **Can you help me**? |
| 3 | **You are a smoking cessation counselor**. I am fifty-six years old and work as a real estate agent. I have been smoking for five years, averaging forty cigarettes a day. The stress from work and the influence of colleagues got me started on smoking. I need to smoke my first cigarette within five minutes of waking up. When drinking with colleagues, I sometimes feel the urge to smoke. I recognize smoking as a bad habit and wish to quit. However, if I do not smoke for twenty-four hours, I find it impossible to concentrate on my work. I am also concerned about gaining weight after quitting. My father and brother smoke, and I live with them. My current girlfriend does not smoke. I wish to start quitting within a week. Normally, I smoke during breaks and after work, especially when going out with colleagues. There are many smokers in my vicinity, and sometimes just passing by them makes it irresistible for me to avoid smoking. **Can you help me**? |
| 4 | **You are a smoking cessation counselor**. I am a forty-five-year-old married individual with a 4-year-old daughter. I have hypertension, which requires medication to manage. I work in construction and smoke about 40 cigarettes daily. I need to smoke my first cigarette within five minutes of waking up. Sometimes, I smoke more when drinking with colleagues after work. I am starting to worry about the negative health effects of smoking. Last week, I attempted to quit smoking but failed due to withdrawal symptoms. I experienced flu-like symptoms, such as headache and fatigue, along with a strong desire to smoke. As a result, I think quitting might be harmful and potentially detrimental to my health, given my long history of smoking. I have never tried nicotine replacement therapy. It's especially hard for me to resist smoking when drinking after work. I don't want to try quitting again. **Can you help me**? |
| 5 | **You are a smoking cessation counselor**. I am a thirty-five-year-old single mother living in a rented apartment with my two lovely children, currently seeking financial assistance. I have been smoking for 10 years and currently smoke 8 cigarettes a day, with my first cigarette sixty minutes after waking up. My family doctor advised me to quit smoking to maintain good health and take care of my children. Although I hope to quit within two months, I am worried that the quitting process might require me to take leave, affecting my income. I often smoke to relax after the children have gone to sleep. However, I realize this habit is harmful to my health and family. My friends suggested I seek smoking cessation counselling for more support and guidance, but due to my financial situation, I hope to find an affordable and effective method to quit smoking. At this critical moment, I need courage and perseverance to change my life and create a healthier future for myself and my children. **Can you help me**? |
| 6 | **You are a smoking cessation counselor**. I am a forty-year-old blue-collar worker living in a less affluent community with my wife and two teenage children. I have been smoking for twenty years and currently smoke fifteen cigarettes a day, needing my first cigarette within thirty minutes of waking up. My doctor has informed me that smoking increases the risk of heart disease and strongly advised me to quit. A recent injury at work has prompted me to consider changing my lifestyle to secure my and my family's future. I aim to quit smoking within three months but am concerned about the high cost of quitting products. Additionally, I worry about the impact quitting may have on my relationships at work, as my colleagues also smoke. To cope with work stress, I often smoke with my colleagues. My wife supports my decision to quit, but as a smoker herself, she might not be able to provide sufficient support. During this challenging time, I need to find the motivation and means to quit smoking to change my life and ensure my family's health. I want to quit smoking. **Can you help me**? |
| 7 | **You are a smoking cessation counselor**. I am a 30-year-old pregnant woman seeking to quit smoking. I am five months pregnant and concerned about the severe harm smoking could cause to my fetus. For the past 10 years, I have maintained a habit of smoking five cigarettes a day, needing my first cigarette sixty minutes after waking up. My doctor has informed me that smoking increases the risks of preterm birth and low birth weight, prompting my decision to quit smoking during pregnancy. However, some of my family members and friends are smokers, and I worry about not receiving their support. They might not understand my need to quit and could even smoke in front of me, presenting a significant temptation. Additionally, I am under immense pressure at home. With my husband unemployed, we are struggling to make ends meet under tight financial conditions. This situation makes me crave smoking as a way to cope with stress, making it even harder to stick to quitting. During this challenging time, I need to find the courage and means to quit smoking to ensure the health of myself and my unborn child. I hope to find a support team to help me overcome this challenge and successfully quit smoking. **Can you help me**? |
| 8 | **You are a smoking cessation counselor**. I am a 45-year-old office worker with type 2 diabetes, hoping to quit smoking within the next six months. I live in a middle-class community with my spouse and two young children. For the past 20 years, I have maintained a habit of smoking ten cigarettes a day, needing my first cigarette within thirty minutes of waking up. My doctor has informed me that smoking could worsen my diabetes and increase the risk of complications. To improve my health, I have decided to quit smoking in the next six months. During this process, I am concerned about the possibility of gaining weight, which could negatively affect my diabetes management. Nevertheless, I understand that quitting smoking is beneficial for my overall health, so I am ready to face this challenge bravely. Fortunately, my spouse is a non-smoker and has provided significant support during my quitting process. With their encouragement, I believe I can overcome this difficulty. During this time, I will seek professional smoking cessation advice and explore how to maintain a stable weight while quitting to better manage my diabetes. I want to try quitting smoking. **Can you help me**? |
| 9 | **You are a smoking cessation counselor**. I am a 23-year-old male university student recently diagnosed with an anxiety disorder, living in a shared apartment with my non-smoking roommate. For the past five years, I have maintained a habit of smoking seven cigarettes a day, with my first cigarette taken sixty minutes after waking up, using smoking as a way to cope with stress and anxiety. However, my therapist has recommended quitting smoking to improve my mental health. While I am motivated to quit, I am concerned about managing my anxiety without cigarettes. Additionally, I worry about the impact quitting may have on my social life, as many of my friends are smokers. I want to try quitting smoking. **Can you help me**? |
| 10 | **You are a smoking cessation counselor**. I am a 67-year-old retiree. I recently experienced a minor stroke, which has prompted me to decide to quit smoking. I live alone in a low-income community where social support is relatively limited. For the past 35 years, I have maintained a habit of smoking twenty cigarettes a day, needing my first cigarette within five minutes of waking up. My doctor has strongly advised me to quit smoking to reduce the risk of having another stroke in the future. Although I am determined to quit, I am concerned about the cost of smoking cessation aids and the lack of social support in my community. My children live far away, but they have been providing me with emotional support over the phone. During this process, I need to find ways to quit smoking with limited social support and under financial strain. **Can you help me**? |
| 11 | **You are a smoking cessation counselor**. I am a 69-year-old self-employed individual with liver disease, hoping to quit smoking. I live in a lower-middle-income community with my two children. For the past 22 years, I have maintained a habit of smoking fourteen cigarettes a day, needing my first cigarette within thirty minutes of waking up. My doctor has told me that quitting smoking is very important for slowing down the progression of liver disease and reducing the risk of complications. Therefore, I have decided to quit smoking within the next four months. However, due to my busy work schedule, I am concerned about how to allocate time and energy for the quitting process. Fortunately, my family supports my decision to quit and is willing to help me through the process. I am initially interested in learning about quitting. **Can you help me**? |
| 12 | **You are a smoking cessation counselor**. I am a 25-year-old single mother hoping to quit smoking. I was recently hospitalized due to a fracture. I live in a low-income community with my two young children. For the past 8 years, I have smoked ten cigarettes a day, needing my first cigarette sixty minutes after waking up. My doctor has told me that quitting smoking is very important for accelerating fracture recovery and preventing future fracture risks. Therefore, I plan to quit smoking within the next three months. To successfully quit, I will seek smoking cessation aids and treatments, such as nicotine replacement therapy or prescription medications. Meanwhile, my children and relatives are willing to provide support during my quitting process. I will rely on their help and encouragement to overcome the challenges of quitting smoking. During the quitting process, I may face challenges with time management and caring for my children. **Can you help me**? |
| 13 | **You are a smoking cessation counselor**. I am a 70-year-old restaurant owner with chronic bronchitis, but I am currently not interested in quitting smoking. I live in a low-income community with my wife and two high school-aged children. For the past 30 years, I have maintained a habit of smoking fifteen cigarettes a day, with a moderate dependence on nicotine, needing my first cigarette within thirty minutes of waking up. My doctor told me that quitting smoking is very important for slowing the progression of chronic bronchitis and improving respiratory symptoms. My family's attitude towards my quitting smoking concerns me, as they wish for me to seek professional help. Although I have not yet decided to quit smoking, I am willing to explore and learn more about the methods and information regarding smoking cessation to make an informed decision in the future. Through this exploration and effort, perhaps I can find a quitting method that suits me, thereby improving my health and bringing comfort to my family. I am not looking to start quitting smoking right now. **Can you help me**? |
| 14 | **You are a smoking cessation counselor**. I am a 13-year-old middle school student struggling with insomnia. Despite having low motivation to quit smoking, I am considering it. I live alone in a middle-income community, smoking five cigarettes a day with a low dependence on nicotine, needing my first cigarette sixty minutes after waking up. My doctor told me that quitting smoking is very important for improving sleep quality and reducing symptoms of insomnia. However, I believe quitting smoking will not significantly impact my quality of life, and I am also concerned that quitting might worsen my insomnia symptoms. My friends are worried about my decision to quit smoking and hope I will seek professional help. I am considering the possibility of quitting smoking in the future. **Can you help me**? |
| 15 | **You are a smoking cessation counselor**. I am a 23-year-old nurse with chronic gastritis, currently experiencing a relapse, and have low motivation to quit smoking. I live in a middle-income community with my husband and a young child. Having smoked for 10 years, I successfully quit for six months but recently relapsed and now smoke eight cigarettes a day, with a low dependence on nicotine, needing my first cigarette sixty minutes after waking up. My doctor told me that quitting smoking is very important for improving symptoms of chronic gastritis and preventing the condition from worsening. However, I believe quitting smoking will not significantly impact my quality of life, and I am also concerned that quitting might increase my stress. My family is worried about my decision to quit smoking and hopes I can seek professional help to quit. **Can you help me**? |
| 16 | **You are a smoking cessation counselor**. I am a 39-year-old construction worker with hyperlipidemia, currently in a relapse phase of quitting smoking, with low motivation to quit. I live in a low-income community with my wife and a middle school-aged child. After smoking for 15 years, I successfully quit for a year but recently relapsed and now smoke 20 cigarettes a day, needing my first cigarette within five minutes of waking up. My doctor told me that quitting smoking is very important for reducing the risk of cardiovascular diseases and improving symptoms of hyperlipidemia. However, I believe that quitting smoking will not significantly impact my quality of life and even worry that quitting might negatively affect my mood. My family and friends are indifferent to my quitting efforts and have not provided enough support. I feel like I cannot successfully quit smoking. **Can you help me**? |
| 17 | **You are a smoking cessation counselor**. I am a 45-year-old entrepreneur considering quitting smoking. I live with my wife and three children and have been smoking for 25 years. Currently, I smoke 15 cigarettes a day, needing the first one within 30 minutes of waking up. Recently, I had a health check-up, and my doctor advised me to quit smoking to reduce the risk of cancer. I hope to quit smoking within six months but am worried about my ability to build relationships in business settings without smoking, as I often smoke during business meetings and social events. However, I realize that quitting smoking is very important for my health and my family, so I have decided to seek professional help. My goal is to find a way to quit smoking that also allows me to maintain my image in business situations. Additionally, I hope to find a support group with other entrepreneurs working to quit smoking. **Can you help me**? |
| 18 | **You are a smoking cessation counselor**. I am a 15-year-old female high school student wishing to quit smoking. I live with my family, who all smoke. I have been smoking for a year now, currently smoking ten cigarettes a day, needing the first one within 30 minutes of waking up. Recently, a relative of mine passed away due to a smoking-related illness, which scared me and made me want to quit. I hope to quit smoking within three months but am worried that I might lose motivation to study afterward. I often smoke to relax while studying. Therefore, I have decided to seek professional help to find the right strategy to quit smoking. **Can you help me**? |
| 19 | **You are a smoking cessation counselor**. I am a 69-year-old lung cancer survivor. I live with my wife and two adult children, and I have been smoking for thirty-five years, currently smoking five cigarettes a day, needing the first one sixty minutes after waking up. After successfully treating lung cancer, my doctor strongly advised me to quit smoking. I hope to quit smoking within a year but am worried about losing social opportunities with friends. As a retiree, I often smoke with old friends in the park. This has been a way for me to relax and connect with friends. The process of trying to quit smoking has been very difficult. I have attempted to quit several times but have always given up due to cravings for cigarettes and fears of losing social activities. My wife and children have been encouraging me to quit smoking, but I still cannot overcome this bad habit. I want to try quitting smoking. **Can you help me**? |
| 20 | **You are a smoking cessation counselor**. I am a 65-year-old farmer, and I do not wish to quit smoking. I live with my two children and have been smoking for thirty years, currently consuming twenty cigarettes a day, needing the first one within five minutes of waking up. Recently, I was hospitalized due to a respiratory disease, and the doctor suggested quitting smoking to improve my health. I hope to quit smoking within a month but am worried about losing contact with friends. In our rural community's activities, I often smoke with friends. As a farmer, I face many socio-economic challenges such as unstable income, intense labor, and lack of medical resources. These issues make me seek ways to decompress after hard work, and smoking has become my method to briefly escape stress. **Can you help me**? |

^a^ The original vignettes were in Traditional Chinese and were translated into English here.
