# Supplementary material for: ChatGPT-4's Consistency, Specificity, and Inclusion of Behavior Change Techniques in Delivering Smoking Cessation Advice in Traditional Chinese: A Content Analysis
Source: Nicotine Tob Res. 2025 Dec 24;28(6):1006–15. doi: 10.1093/ntr/ntaf267 (PMC13196701; doi:10.1093/ntr/ntaf267)
Supplement: Supplementary_Material_5_ntaf267(1) [file supplementary_material_5_ntaf267(1).docx]

# Supplementary Material 5. Adapted BCTs checklist for phase 3

| **BCT Group** | **Label** | **Reason for exclusion** |
| --- | --- | --- |
| BM | BM1 Provide information on consequences of smoking and smoking cessation |  |
|  | BM2 Boost motivation and self-efficacy |  |
|  | BM3 Provide feedback on current behavior | Not applicable because it refers to feedback on progress towards becoming a permanent non-smoker, whereas in this study, the 'smoker' is just beginning to seek help |
|  | BM4 Provide rewards contingent on successfully stopping smoking | Not applicable because the 'smoker' is just beginning to seek help |
|  | BM5 Provide normative information about others' behavior and experiences | Not delivered |
|  | BM6 Prompt commitment from the client there and then | Not delivered |
|  | BM7 Provide rewards contingent on effort or progress | Not applicable because no progress has been made as the 'smoker' is just beginning to seek help and yet to quit |
|  | BM8 Strengthen ex-smoker identity | Not applicable because the 'smoker' is just beginning to seek help and yet to quit |
|  | BM9 Identify reasons for wanting and not wanting to stop smoking |  |
|  | BM10 Explain the importance of abrupt cessation | Not delivered |
|  | BM11 Measure CO | Not applicable because ChatGPT cannot measure CO |
| BS | BS1 Facilitate barrier identification and problem solving |  |
|  | BS2 Facilitate relapse prevention and coping |  |
|  | BS3 Facilitate action planning/develop treatment plan |  |
|  | BS4 Facilitate goal setting |  |
|  | BS5 Prompt review of goals | Not applicable because the 'smoker' is just beginning to seek help and does not have a goal to review yet |
|  | BS6 Prompt self-recording | Not delivered |
|  | BS7 Advise on changing routine |  |
|  | BS8 Advise on environmental restructuring |  |
|  | BS9 Set graded tasks |  |
|  | BS10 Advise on conserving mental resources | Not delivered |
|  | BS11 Advise on avoiding social cues for smoking |  |
| A | A1 Advise on stop-smoking medication |  |
|  | A2 Advise on/facilitate use of social support |  |
|  | A3 Adopt appropriate local procedures to enable clients to obtain free medication | Not applicable because ChatGPT cannot help the smoker get his/her medication easily and without charge |
|  | A4 Ask about experiences of stop smoking medication that the smoker is using | Not delivered |
|  | A5 Give options for additional and later support |  |
| RD | RD1 Tailor interactions appropriately |  |
|  | RD2 Emphasize choice | Not delivered |
| RI | RI1 Assess current and past smoking behavior | Not applicable because there has only been one round of conversation, initiated by the smoker, ChatGPT cannot proactively assess this information and can only respond |
|  | RI2 Assess current readiness and ability to quit | Not applicable because there has only been one round of conversation, initiated by the smoker, ChatGPT cannot proactively assess this information and can only respond |
|  | RI3 Assess past history of quit attempts | Not applicable because there has only been one round of conversation, initiated by the smoker, ChatGPT cannot proactively assess this information and can only respond |
|  | RI4 Assess withdrawal symptoms | Not applicable because there has only been one round of conversation, initiated by the smoker, ChatGPT cannot proactively assess this information and can only respond |
| RC | RC1 Build general rapport |  |
|  | RC2 Elicit and answer questions | Not applicable because there has only been one round of conversation, initiated by the smoker, ChatGPT cannot elicit questions from the smoker |
|  | RC3 Explain the purpose of CO monitoring | Not applicable because ChatGPT has not measured CO and does not need to explain the purpose |
|  | RC4 Explain expectations regarding treatment programme | Not delivered |
|  | RC5 Offer/direct towards appropriate written materials | Not applicable because ChatGPT does not possess any written material to be offered or directed to smokers |
|  | RC6 Provide information on withdrawal symptoms |  |
|  | RC7 Use reflective listening | Not applicable because ChatGPT can only respond after receiving information and there has been only one round of conversation, it cannot reflect back to the smoker |
|  | RC8 Elicit client views | Not applicable because there has only been one round of conversation, initiated by the smoker, ChatGPT cannot elicit views from the smoker |
|  | RC9 Summarize information/confirm client decisions | Not applicable because the 'smoker' is just beginning to seek help and has not yet reached the stage of summarizing information and confirming decision |
|  | RC10 Provide reassurance | Not delivered |

*Note:* BCTs in grey were deleted because they were not applicable in this study or were never delivered in any piece of ChatGPT advice.

BM: Specific focus on behavior (B) and addressing motivation (M); BS: Specific focus on behavior (B) and maximize self-regulatory capacity/skill (S); A: Promote adjuvant activities; RD: General aspects of the interaction (R) focusing on delivery of the intervention (D); RC: General aspects of the interaction (R) focusing on general communication (C)
