# Supplementary material for: ChatGPT-4's Consistency, Specificity, and Inclusion of Behavior Change Techniques in Delivering Smoking Cessation Advice in Traditional Chinese: A Content Analysis
Source: Nicotine Tob Res. 2025 Dec 24;28(6):1006–15. doi: 10.1093/ntr/ntaf267 (PMC13196701; doi:10.1093/ntr/ntaf267)
Supplement: Supplementary_Material_6_ntaf267(1) [file supplementary_material_6_ntaf267(1).docx]

# Supplementary Material 6. Phase 3 coding manual

## ChatGPT Smoking Cessation Suggestion Behavior Change Techniques (BCTs) Coding Manual

### Introduction

Our study aims to evaluate the performance of ChatGPT on providing smoking cessation related suggestions. 20 scenarios containing detailed smoking-related information were inputted into ChatGPT with a GPT-4 default model to generate corresponding responses. In order to summarize the effective components in a systematic and theory-based manner, Behavior Change Techniques Taxonomy of behavioral support for smoking cessation (BCTTsm) was utilized as the template and theoretical foundation to code them by outlining Behavior Change Techniques (BCTs) occurred in each response. This manual serves as a comprehensive guide for accurately coding BCTs in the context of ChatGPT's Smoking Cessation Suggestion, ensuring consistency and precision in the analysis of BCTs.

### Background Information

#### Overview of ChatGPT and its Smoking Cessation Suggestion

ChatGPT, which stands for Chat Generative Pre-trained Transformer, is a large language model-based chatbot developed by OpenAI and launched on November 30, 2022^1^. With its ability to generate human-like text based on large amounts of data, ChatGPT has the potential to be applied in the healthcare and medical domains^2,3^. Most studies examined ChatGPT’s performance on relevant standardized examinations, several studies showed that GPT 4.0 can pass standard exams related to medicine^4–6^, and one even found that it can achieve expert-level performance in the exam^7^, suggesting a promising prospect of assisting with medical education, and potentially, clinical decision-making. Practical ChatGPT applications in this field involve assisting clinical diagnosis^8^, providing clinical decision support^9,10^, and answering basic medication consultation questions^11^. However, there is a lack of studies examining the usefulness of ChatGPT for smoking cessation intervention. After searching in PubMed, Google Scholar, and Web of Science, we resulted in only one study exploring ChatGPT’s usefulness in vaping cessation concluding that the ChatGPT responses performed well in terms of accuracy, quality, clarity, and empathy^12^, and this study is conducted in English context, not Chinese.

#### Overview of Behavior Change Techniques (BCTs)

Behavior Change Techniques (BCTs) are a set of evidence-based strategies or methods employed in the field of behavioral psychology and health promotion to facilitate desired changes in individual behavior. It was first developed by Abraham and Michie in 2008 to specify and report intervention components^13^. BCT means an observable, replicable, and irreducible component of an intervention designed to alter or redirect causal processes that regulate behavior^14^. In other words, a BCT is proposed to be an “active ingredient” of intervention. Effective BCTs have been identified for interventions to diverse health behavior, including smoking cessation^15–18^.

### BCT taxonomy of behavioral support for smoking cessation (BCTTsm) Overview

#### Overview of BCT Taxonomy

BCT Taxonomies are comprehensive and systematic frameworks used in the field of behavioral science and health promotion to categorize and define specific techniques that influence human behavior. A BCT taxonomy provides a standardized way of identifying, describing, and coding various strategies and interventions aimed at changing a specific behavior. This method has been widely used to report interventions, synthesize evidence, and design interventions. BCT Taxonomies were typically developed by synthesizing relevant comprehensive intervention materials based on different target behaviors and domains with the input of expert consensus. One of the most comprehensive and widely-used BCT Taxonomies is the Behavior Change Technique Taxonomy (v1)^14^, a hierarchically structured taxonomy of techniques consisting of 93 BCTs.

#### Definitions and Categories of BCTTsm

BCTTsm is a taxonomy of BCTs used within individual behavioral support for smoking cessation developed by Michie in 2011^17^. This taxonomy was generated after extensive analysis of two key source documents and was further refined through practical coding. BCTTsm is comprised of forty-three BCTs which could be classified into four functions: 1) directly addressing motivation e.g. providing rewards contingent on abstinence, 2) maximizing self-regulatory capacity or skills e.g. facilitating barrier identification and problem solving, 3) promoting adjuvant activities e.g. advising on stop-smoking medication, and 4) supporting other BCTs e.g. building general rapport. (See the appendix for the complete BCTTsm) This taxonomy is selected as the template and theoretical foundation to code ChatGPT Smoking Cessation Suggestions as it provides a relevant and structural framework for systematically identifying and categorizing the behavior change techniques used within this specific context.

### Coding Guidelines

The typical format for responses includes an introductory or greeting paragraph, several bullet points of recommendations, and a concluding summary paragraph. The BCTTsm provides a detailed description of each BCT, coders are suggested to have an overview of this manual and the BCTTsm before coding and code each paragraph and bullet point verbatim, avoiding subjective interpretations go astray from this manual and the BCTTsm.

A complete BCTTsm is attached for coders’ reference, BCTs deemed not relevant to this analysis in the preliminary coding are highlighted in grey, but coders are still encouraged to check out these BCTs in case any BCT was overlooked.

It should be noted that one paragraph or bullet point can involve more than one BCT and the BCTs within BCTTsm may also fail to capture the essence of it (in this scenario, leave this paragraph or bullet point uncoded).

### Application Scenarios

Relevant BCTs of each type of suggestion bullet point were presented below with explanations for its coding.

**Suggestion bullet point 1:**

戒煙是一個很好的決定，對你的健康有很大的益處 (*Quitting smoking is a great decision and has significant benefits for your health.*) **^*^**

**BCTs coded:**

BM1 Provide information on consequences of smoking and smoking cessation (Mentions the health benefit of quitting smoking)^**^

BM2 Boost motivation and self-efficacy (Give encouragement for the recipient’s decision on quitting smoking)

**Suggestion bullet point 2:**

制定戒煙計劃：選擇一個戒煙的開始日期, 並告訴家人和朋友你的決定，以便他們給予支持。(*Developing a Smoking Cessation Plan: Choose a start date to quit smoking and inform your family and friends about your decision so they can offer support.*)

**BCTs coded:**

BS4 Facilitate goal setting (Help the smoker to set a quit date)

A2 Advise on/facilitate use of social support (Facilitate utilization of family and social support)

**Suggestion bullet point 3:**

使用尼古丁替代品：尼古丁替代療法，例如尼古丁貼片、口香糖或含有尼古丁的吸入器，可以幫助緩解戒煙時的尼古丁渴求 (*Using Nicotine Replacements: Nicotine replacement therapy, such as nicotine patches, gum, or inhalers containing nicotine, can help alleviate nicotine cravings during smoking cessation.*)

**BCTs coded:**

A1 Advise on stop-smoking medication (Mentions the use of medication)

**Suggestion bullet point 4:**

改變生活習慣：試著找出引起你吸煙的原因，例如壓力、悶熱或與某些朋友在一起。然後，嘗試改變這些習慣，以減少吸煙的誘惑。(*Changing Lifestyle Habits: Try to identify the triggers that lead you to smoke, such as stress, boredom, or being with certain friends. Then, attempt to change these habits to reduce the temptation to smoke.*)

**BCTs coded:**

BS1 Facilitate barrier identification and problem solving (Help the smoker to develop general ways of addressing barriers of cessation)

BS7 Advise on changing routine (Mentioned changing habit)

BS11 Advise on avoiding social cues for smoking (Mentions friends as a cue for smoking)

**Suggestion bullet point 5:**

保持忙碌：尋找其他活動來取代吸煙，例如運動、學習新技能或參加社交活動 (*Stay Busy: Look for alternative activities to replace smoking, such as exercising, learning new skills, or participating in social events.*)

減壓 - 找到其他方法來應對壓力，例如瑜伽、冥想或呼吸運動 (*Reduce Stress: Find other ways to cope with stress, such as yoga, meditation, or breathing exercises.*)

運動 - 增加運動量可以幫助您減少對香煙的渴望，同時改善您的健康狀況 (*Exercise: Increasing your physical activity can help reduce your cravings for cigarettes while also improving your overall health*.)

**BCTs coded:**

BS1 Facilitate barrier identification and problem solving (Help the smoker to develop general ways of addressing barriers of cessation)

**Suggestion bullet point 6:**

獎勵自己：每當達到一個戒煙的里程碑，例如戒煙一周、一個月或一年，給自己一個獎勵以激勵持續戒煙 (*Reward Yourself: Whenever you reach a smoking cessation milestone, such as one week, one month, or one year without smoking, give yourself a reward to motivate continued success in quitting smoking.*)

**BCTs coded:**

BS3 Facilitate action planning/develop treatment plan (Rewarding oneself is part of a quitting plan?)

**Suggestion bullet point 7:**

不要灰心：戒煙過程中可能會有復吸的情況，不要灰心，繼續堅持戒煙，並從中學習如何避免再次復吸。 (*Don't Get Discouraged: Relapses may occur during the smoking cessation process. Don't be disheartened. Continue your efforts to quit and learn from the experience to avoid future relapses.*)

**BCTs coded:**

BS2 Facilitate relapse prevention and coping (Help the smoker to prepare for lapses and avoiding lapses turning into relapse)

**Suggestion bullet point 8:**

吸煙對您的健康有很大的危害，特別是在您這麼年輕的年齡。(*Smoking poses significant health risks, especially at such a young age.*)

**BCTs coded:**

RD1 Tailor interactions appropriately (identify specific information other readiness to quit, in this case, it mentions the user’s age, this is a sign of targeting the user's characteristics and tailoring its response, though not satisfying.)

**Suggestion bullet point 9:**

當然可以幫你！戒煙是一個很好的決定，對你的健康有很大的益處。以下是一些建議，希望對你戒煙有所幫助： (*Of course, I can help you! Quitting smoking is a great decision and offers significant health benefits. Here are some suggestions that might help you with your smoking cessation:…*)

希望這些建議對你有所幫助，祝你戒煙成功！(*I hope these suggestions are helpful to you, and I wish you success in quitting smoking!*)

**BCTs coded:**

RC1 Build general rapport (Establishes a positive, friendly and professional relationship with the smoker throughout the response)

**Suggestion bullet point 10:**

確定戒煙的理由：將這些理由記下來，並在需要動力時提醒自己。(*Identify Your Reasons for Quitting: Write these reasons down and remind yourself of them when you need motivation.*)

**BCTs coded:**

BM9 Identify reasons for wanting and not wanting to stop smoking (Help the smoker determine why it is important to stop)

**Suggestion bullet point 11:**

尋求專業幫助：戒煙輔導、專業醫生或藥物治療都可以提高戒煙成功的機會(*Seek Professional Help: Smoking cessation counselling, consulting with a medical professional, or pharmacotherapy can all increase the chances of successfully quitting smoking.*)

**BCTs coded:**

A5 Give options for additional and later support (Mentions additional professional assistance)

**Suggestion bullet point 12:**

了解戒煙症狀：戒煙過程中，您可能會經歷一些戒斷症狀，如焦慮、煩躁和食慾增加。了解這些症狀有助於您更好地應對它們。(*Understand Withdrawal Symptoms: During the smoking cessation process, you may experience some withdrawal symptoms such as anxiety, irritability, and increased appetite. Understanding these symptoms can help you better cope with them.*)

**BCTs coded:**

RC6 Provide information on withdrawal symptoms (Mentions withdrawal symptoms)

**Suggestion bullet point 13:**

嘗試減少吸煙次數：您可以嘗試逐步減少每天吸煙的次數，從而減少對健康的影響。例如，您可以試著將每天的煙量從15枝減少到10枝 (*Try Reducing the Number of Cigarettes: You can attempt to gradually reduce the number of cigarettes you smoke each day to lessen the impact on your health. For example, you could try reducing your daily consumption from 15 cigarettes to 10.*)

**BCTs coded:**

BS9 Set graded tasks (Suggests small achievable goals)

**Suggestion bullet point 14:**

遠離誘惑：避免與吸煙者在一起的場合，將家中和車內的煙草產品清除乾淨 (*Avoid Temptations: Stay away from situations where you are around smokers, and clean out all tobacco products from your home and car.*)

**BCTs coded:**

BS8 Advise on environmental restructuring (Mentions changing the physical environment)

BS11 Advise on avoiding social cues for smoking (Mentions other smokers as a smoking cue)

^*^: The original examples in this section were in Traditional Chinese because the responses generated by ChatGPT were in Traditional Chinese, we translated it into English for the readers of this paper for easier understanding.

^**^: The content in red within the brackets serves as the justification for coding this example into this BCT.

### Quality Assurance

Two coders with expertise in smoking cessation will code the ChatGPT Smoking Cessation Suggestions based on the BCTTsm independently after training. In instances of coding disagreements, a third researcher will intervene to independently recode the inconsistent parts and serves as an arbitrator.

When coders encounter situations that are difficult to judge or where coding criteria are ambiguous, inquiry could be directed to the third researcher at [xxy167@connect.hku.hk](mailto:xiexiaoyun0605@163.com) through email.

### References

1. ChatGPT. In: *Wikipedia*. ; 2023. Accessed October 23, 2023. https://en.wikipedia.org/w/index.php?title=ChatGPT&oldid=1181235235

2. Dave T, Athaluri SA, Singh S. ChatGPT in medicine: an overview of its applications, advantages, limitations, future prospects, and ethical considerations. *Front Artif Intell* 2023;**6:**1169595.

3. Biswas SS. Role of Chat GPT in Public Health. *Ann Biomed Eng* 2023;**51(5):**868-869.

4. Kung JE, Marshall C, Gauthier C, Gonzalez TA, Jackson JB. Evaluating ChatGPT Performance on the Orthopaedic In-Training Examination. *JB JS Open Access* 2023;**8(3):**e23.00056.

5. Kung TH, Cheatham M, Medenilla A, et al. Performance of ChatGPT on USMLE: Potential for AI-assisted medical education using large language models. *PLOS Digit Health* 2023;**2(2):**e0000198.

6. Wójcik S, Rulkiewicz A, Pruszczyk P, Lisik W, Poboży M, Domienik-Karłowicz J. Reshaping medical education: Performance of ChatGPT on a PES medical examination. *Cardiol J*. Published online October 13, 2023. doi:10.5603/cj.97517

7. Flores-Cohaila JA, García-Vicente A, Vizcarra-Jiménez SF, et al. Performance of ChatGPT on the Peruvian National Licensing Medical Examination: Cross-Sectional Study. *JMIR Med Educ* 2023;**9:**e48039.

8. Madadi Y, Delsoz M, Lao PA, et al. ChatGPT Assisting Diagnosis of Neuro-ophthalmology Diseases Based on Case Reports. *MedRxiv Prepr Serv Health Sci*. Published online September 14, 2023:2023.09.13.23295508. doi:10.1101/2023.09.13.23295508

9. Rao A, Kim J, Kamineni M, Pang M, Lie W, Succi MD. Evaluating ChatGPT as an Adjunct for Radiologic Decision-Making. *MedRxiv Prepr Serv Health Sci*. Published online February 7, 2023:2023.02.02.23285399. doi:10.1101/2023.02.02.23285399

10. Yeo YH, Samaan JS, Ng WH, et al. Assessing the performance of ChatGPT in answering questions regarding cirrhosis and hepatocellular carcinoma. *Clin Mol Hepatol* 2023;**29(3):**721-732.

11. Hsu HY, Hsu KC, Hou SY, Wu CL, Hsieh YW, Cheng YD. Examining Real-World Medication Consultations and Drug-Herb Interactions: ChatGPT Performance Evaluation. *JMIR Med Educ* 2023;**9:**e48433.

12. Amin S, Kawamoto CT, Pokhrel P. Exploring the ChatGPT platform with scenario-specific prompts for vaping cessation. *Tob Control*. Published online July 17, 2023:tc-2023-058009. doi:10.1136/tc-2023-058009

13. Abraham C, Michie S. A taxonomy of behavior change techniques used in interventions. *Health Psychol Off J Div Health Psychol Am Psychol Assoc* 2008;**27(3):**379-387.

14. Michie S, Richardson M, Johnston M, et al. The behavior change technique taxonomy (v1) of 93 hierarchically clustered techniques: building an international consensus for the reporting of behavior change interventions. *Ann Behav Med Publ Soc Behav Med* 2013;**46(1):**81-95.

15. West R, Walia A, Hyder N, Shahab L, Michie S. Behavior change techniques used by the English Stop Smoking Services and their associations with short-term quit outcomes. *Nicotine Tob Res Off J Soc Res Nicotine Tob* 2010;**12(7):**742-747.

16. Black N, Johnston M, Michie S, et al. Behaviour change techniques associated with smoking cessation in intervention and comparator groups of randomized controlled trials: a systematic review and meta-regression. *Addict Abingdon Engl* 2020;**115(11):**2008-2020.

17. Michie S, Hyder N, Walia A, West R. Development of a taxonomy of behaviour change techniques used in individual behavioural support for smoking cessation. *Addict Behav* 2011;**36(4):**315-319.

18. Michie S, Abraham C, Whittington C, McAteer J, Gupta S. Effective techniques in healthy eating and physical activity interventions: a meta-regression. *Health Psychol Off J Div Health Psychol Am Psychol Assoc* 2009;**28(6):**690-701.

*Note:* The BCT taxonomy of behavioral support for smoking cessation (BCTTsm) was also attached in this manual for coders’ reference, it was removed to ensure the conciseness of this appendix.
